# Lesion of the dopaminergic nigrostriatal pathway induces trigeminal dynamic mechanical allodynia

**DOI:** 10.1002/brb3.214

**Published:** 2014-02-20

**Authors:** Wisam Dieb, Omar Ouachikh, Franck Durif, Aziz Hafidi

**Affiliations:** 1 Laboratoire de neuro-psychopharmacology des systèmes dopaminergiques sous corticaux, Clermont Université, Université d'AuvergneClermont-Ferrand, EA7280, France; 2 Service de Neurologie, CHU Clermont-FerrandClermont-Ferrand, 63000, France

**Keywords:** Atypical facial algia, basal ganglia, burning mouth syndrome, orofacial pain, Parkinson disease, trigeminal subnucleus caudalis (Sp5C)

## Abstract

**Background:**

Pain constitutes the major non motor syndrome in Parkinson's disease (PD) and includes neuropathic pain; however current drug therapies used to alleviate it have only limited efficacy. This is probably due to poor understanding of the mechanisms underlying it.

**Aims:**

We investigated a major class of trigeminal neuropathic pain, dynamic mechanical allodynia (DMA), in a rat model of PD and in which a bilateral 6-hydroxy dopamine (6-OHDA) injection was administered to produce a lesion of the nigrostriatal dopaminergic pathway.

**Results and discussion:**

Lesioned animals presented significant DMA in the orofacial area that occurred from 4 days to 5 weeks post-injury. To investigate a segmental implication in the neuropathic pain induced by dopamine depletion, the expression of the isoform gamma of the protein kinase C (PKCg) and phosphorylated extracellular signal-regulated kinases 1/2 (pERK1/2) was explored in the medullary dorsal horn (MDH). There was a high increase in PKCg expression in the III and IIi laminae of the MDH of lesioned-animals compared to shams. pERK1/2 expression was also significantly high in the ipsilateral MDH of lesioned rats in response to non-noxious tactile stimulus of the orofacial region. Since pERK1/2 is expressed only in response to nociceptive stimuli in the dorsal spinal horn, the current study demonstrates that non-noxious stimuli evoke allodynic response. Intraperitoneal and intracisternal administrations of bromocriptine, a dopamine 2 receptor (D2R) agonist, significantly decreased DMA compared to control rats injected with saline. These data demonstrate for the first time that nigrostriatal dopaminergic depletion produces trigeminal neuropathic pain that at least involves a segmental mechanism. In addition, bromocriptine was shown to have a remarkable analgesic effect on this neuropathic pain symptom.

## Introduction

Dopamine (DA) dysfunction is implicated in the modulation of pain perception and analgesia (Chudler and Dong 1995; Wasner and Deuschl 2012) and DA depletion plays a central role in this modulation. Indeed, hyposensitivity to pain is common in patients with schizophrenia, which is linked to excessive DA neurotransmission. Conversely, hypersensitivity to pain is frequent in disorders linked to DA dysregulation such as mood and affect disorders, burning mouth, and Parkinson's disease (PD). In PD, DA depletion in target areas provokes progressive motor disabilities, and cognitive and vegetative disturbances (Lin et al. 1981; Clifford et al. 1998; Fischer et al. 2005). PD is also characterized by nonmotor manifestations (NMM), which may precede or occur during the onset of motor disturbances (Pertovaara et al. 2004). One of the NMM in PD is pain (Cobacho et al. 2010; Goetz 2011; Ha and Jankovic 2012) and epidemiological studies have estimated its prevalence in PD to be 30–83% (Barceló et al. 2010; Wasner and Deuschl 2012). Preclinical studies using different paradigms have implicated basal ganglia in pain processes (Chudler and Dong 1995; Wood 2006; Chudler and Lu 2008; Borsook 2012). For example, DA depletion in the striatum leads to an increase in neuropathic pain (Saadé et al. 1997). Conversely, an enhancement of DA release by amphetamine infusion into the nucleus accumbens facilitates the inhibition of tonic pain (Altier and Stewart 1999).

Neuropathic pain is clinically characterized by spontaneous pain and evoked pain. It can result from the primary dysfunction of the peripheral nociceptive and nonnociceptive nerves of the central nervous system (Rizvi et al. 1991). Unfortunately, the treatment of neuropathic pain is often unsatisfactory, mostly due to the limited efficacy of currently available drug therapies. Touch-evoked pain is a hallmark of allodynia, and is generally considered to result from the activation of large myelinated A-fibers, which normally convey nonnoxious mechanical stimulation (Campbell et al. 1988; Ochoa and Yarnitsky 1993; Koltzenburg et al. 1994; Sandkühler 2009). After nerve injury, tactile stimulation is able to evoke dynamic mechanical allodynia (DMA), which can be elicited by light moving stimuli (i.e., stroking or light brushing) of the skin (Woolf and Mannion 1999; Alvarez et al. 2009; Miraucourt et al. 2009). Air puffs or jets have been shown to activate preferentially low-threshold A*β*-fibers, constituting a useful tool for investigating DMA (Sandkühler 2009).

The spinal cord is an important gateway through which peripheral pain signals are transmitted to the brain. Spinal sensitization is one of the main mechanisms underlying neuropathic pain (Woolf and Mannion 1999). Two markers were used, namely protein kinase C (PKC*γ*) a stress sensor protein, and phosphorylated forms of ERK1/2, to demonstrate medullary dorsal horn (MDH) (equivalent of spinal dorsal horn) sensitization at both cellular and molecular levels. Within the superficial dorsal horn, PKC*γ* is restricted to a subpopulation of interneurons in the inner part of lamina II (IIi) (Malmberg et al. 1997; Polgár et al. 1999). Its activation is involved in hyperexcitability, persistent pain states, and the transition from short to long-term hyperexcitability (Malmberg et al. 1997; Martin et al. 1999; Miletic et al. 2000; Ohsawa et al. 2001; Wang et al. 2005; Nakajima et al. 2011).

ERK1/2 phosphorylation constitutes a selective cell marker which occurs in response to noxious stimulus (Ji et al. 1999) and not to nonnoxious stimulus such as light touch.

This study first aimed at determining whether nigrostriatal dopamine depletion could induce trigeminal DMA in the rat. We used an animal model for PD (Paillé et al. 2007; Zengin-Toktas et al. 2013). In this model, lesions within the substantia nigra compacta (SNc) and ventral tegmental area (VTA) were obtained by injecting the 6-OHDA into the medial forebrain bundle. Second, we asked whether a local segmental mechanism is implicated in this type of allodynia. Finally, we investigated whether the action of bromocriptine, a dopamine 2 receptor (D2R) agonist drug, has analgesic effects in this animal model of PD.

## Materials and Methods

### Ethical statement

The experiments conformed to the ethical guidelines of the International Association for the Study of Pain, the European Community Council directive of 24 November 1986 (86/609/EEC) and the Animal Ethics Committee of the University of Auvergne (CE08-11). All surgery was performed under anesthesia, and every effort was made to minimize animal suffering and number. The rats were kept in specified pathogen-free conditions, and all the procedures performed were approved by the Auvergne University ethics committee.

### Surgery

Eighty-two adult male Sprague–Dawley rats (275–325 g) from Charles River (L'Arbresle, France) were obtained and maintained in a controlled environment (lights on 07:00–19:00, 22°C) with ad libitum access to food and water. They were housed three to four per cage.

The experiment was performed as described previously (Paillé et al. 2007; Zengin-Toktas et al. 2013). Anesthesia was given with ketamine 60 mg/kg, i.p. and Rompun® (Bayer, France) (xylazine, 10 mg/kg, i.p.). The animals were placed in a stereotaxic frame (David Kopf Instrument, Tujunga, CA). Eighty-two rats were injected bilaterally in the substantia nigra compacta (SNc) with 6-OHDA (6-hydroxy dopamine, 0.5 *μ*L/min) after dissolution in a vehicle solution (0.02% ascorbate saline) at a concentration of 3 *μ*g/*μ*L (Sigma-Aldrich, Saint-Quentin, France) in two deposits (2.25 and 2.85 *μ*g, respectively) at the following coordinates (in mm relative to bregma and the surface of the dura mater): posterior (P) −4.0; lateral (L) ± 0.8; ventral (V) −8.0; tooth bar at +3.4 and A −4.4; L ± 1.2; V −7.8; tooth bar at −2.4. In order to preserve adrenergic neurons from 6-OHDA toxicity, the animals received desipramine (25 mg/kg, i.p., Sigma-Aldrich) 30 min prior to the toxin injection; sham-lesioned rats received only the vehicle at the same coordinates.

### Drugs

The following drugs were used: bromocriptine (Sigma-Aldrich) dissolved in 0.9% saline, and sulpiride (Sigma-Aldrich) dissolved in 2.5% HCL, 7.5% dimethyl sulfoxide (DMSO), 90% saline (0.9%). Fresh solutions were prepared just prior to use. In line with our previous study (Zengin-Toktas et al. 2013), a concentration of bromocriptine at 1 mg/kg was selected for i.p. injection and at 7 *μ*g/kg for intracisternal injection. Sulpiride was used at 50 mg/kg (Melo et al. 2013).

### Drugs administration

The analgesic effects of bromocriptine, a drug used as PD therapy (Calne et al. 1974), were studied by injecting the drug both intracisternally (Fischer et al. 2005) and intraperitoneally. Bromocriptine is known to cross the blood brain barrier (Vautier et al. 2009). The animals were briefly (<3 min) anesthetized with 2% halothane using a mask and received intracisternal administration of bromocriptine (7 *μ*L/kg dissolved in 5 *μ*L vehicle) or the vehicle alone (5 *μ*L of 0.9% saline). Following recovery (<2 min), the rats were placed in the observation field under a red light for a 40-min test period. Gentle air puffing (1 sec duration) was applied every 3 min (Alvarez et al. 2009).

For the intraperitoneal injection, bromocriptine was used at a dose of 1 mg/kg and its effect was studied for a 6-h test period.

Finally, the antagonist action of sulpiride on the bromocriptine-induced analgesic effect was assessed by administrating this compound (50 mg/kg) 90 min prior to the intracisternal injection of bromocriptine.

### Behavioral testing

The rats were adapted to the observation field and red light for 30 min each day for 9 days prior to the beginning of behavioral testing. During this period, the experimenter reached into the cage to apply gentle air puffing on the animals' faces (see below). For each behavioral test, the rats were placed in the observation field (24 × 35 × 18 cm) under red light for a 30-min period. Gentle air puffing (five air puffs, with a 5-sec time lag between each air puff) was applied by an experimenter (Dieb and Hafidi 2013) every 3 min and repeated 10 times, alternatively on either side of the vibrissal pad, using a calibrated pump. Stimulation was carried out when the rat was in a sniffing/no locomotion state: with four paws placed on the ground, neither moving nor freezing (Alvarez et al. 2009). The distance to the target from which the stimulus was applied varied from 2 to 5 cm. The tip of the pump was moved toward the target from behind the animal so that it could not see it. Each series of stimulations consisted of five air puffs applied every 10 sec.

The behavioral responses were observed and quantified according to the analysis method proposed by Vos et al. (1994). Nociceptive responses to gentle air puffing consisted of one or more of the following elements: (Altier and Stewart 1999) detection, the rats turn head toward stimulus; (Alvarez et al. 2009) withdrawal reaction, the rats turn head away or pull it briskly backward when stimulation is applied (a withdrawal reaction is assumed to include a detection element preceding head withdrawal and therefore consists of two response elements); (Ansah et al. 2007) escape/attack, the rats avoid further contact with the stimulus, either passively by moving their body away from the stimulus, or actively by attacking the tip of the pump; (Barceló et al. 2010) asymmetric grooming, the rats display an uninterrupted series of at least three wash strokes directed to the stimulated area. The score was assumed to reflect the magnitude of the aversiveness evoked by the mechanical stimulation, being equal to zero in the case of absence of response. A mean score value was then calculated for each stimulation series.

### Immunohistochemical analysis

The rats were deeply anesthetized with urethane (1.5 g/kg i.p.). Twenty minutes after the induction of anesthesia, rats were stimulated for 2 min on one infraorbital region by gentle air puffing (60 stimuli delivered, 0.5 Hz). Three minutes after the end of stimulation, the rats were perfused transcardially with warm (37°C) heparinized saline (25 IU heparin/mL) followed by cold (10°C) phosphate-buffered solution (0.1 mol/L, pH 7.6) containing 4% paraformaldehyde and 0.03% picric acid for 15 min. The brainstem was then removed and transferred to the same fixative solution for 1 h and then placed in 30% sucrose and 0.05% sodium azide solution overnight at 4°C. Coronal 40-*μ*m thick sections of the brainstem were cut on a freezing microtome (Leica, Wetzlar, Germany) and collected in a 0.05 mol/L Tris-buffered saline (TBS).

For immunofluorescence, free-floating brainstem sections were placed in 1% normal goat serum for 1 h before overnight incubation at room temperature in primary antibody solutions (mouse and rabbit antiphosphorylated extracellular signal-regulated kinases 1/2 [pERK1/2] [1:1000, Cell Signaling Technologies, Danvers, MA], and mouse and rabbit anti-PKC*γ* [1:4000, Sigma-Aldrich and Santa Cruz, Dallas, TX]). The corresponding secondary antibodies (1:400 for goat anti-mouse Cy3, 1:200 for goat anti-rabbit Cy2) were incubated at room temperature for 3 h. All antibodies were diluted in TBS containing 0.25% bovine serum albumin and 0.3% Triton X-100. The sections were finally rinsed in TBS, mounted on gelatin-coated slides, dehydrated in alcohol, cleared in xylene, and coverslipped with 1,3-diethyl-8-phenylxanthine (DPX) medium. The specificity of the immunostaining was assessed by omitting the primary antibodies, which resulted in the absence of signal.

Immunofluorescent staining was analyzed by using a motorized Zeiss Axioplan 2 microscope equipped with a Hamamatsu C4742-95 digital camera (Hamamatsu Photonics France SARL, Massy, France) (switching between FITC and Texas Red filter sets) driven by MetaMorph® 5.4 software. In each rat, image acquisition and fluorescent signal quantification were done using seven different sections, each taken at a given rostrocaudal plane within the MDH (from 0 to −2160 *μ*m at 360 *μ*m intervals). To reduce the variability in staining between sections, the same area of the MDH was taken in consideration for quantification of the fluorescent signal. Measurement of PKC*γ* fluorescence was taken from the layer IIi as it contained the majority of interneurons positive for this marker. The data are collected from seven sections in each rat. The final data are presented as a mean value of the total number of each rat group. Brainstem sections were categorized according to their approximate rostrocaudal location from the MDH subnucleus interpolaris junction. The pERK1/2-positive cells were counted under a 20× objective according to their location in the different laminae of the MDH from sections costained for PKC*γ*, a cellular marker that highlights the inner lamina II (Polgár et al. 1999). The delineation of the MDH was based on the Paxinos and Watson atlas. The data are expressed as the sum of the total number of labeled cells counted from all the sections analyzed in each animal.

Tyrosine hydroxylase (TH) immunolabeling was performed using anti-TH primary antibody (Millipore, Molsheim, France), as described above. Quantification of the impact of the 6-OHDA lesion on the SNc was investigated as described previously (Paillé et al. 2007; Zengin-Toktas et al. 2013) using frozen coronal sections (40 *μ*m) under a 20× objective. For each rat, TH-positive cells were counted at different rostrocaudal levels (every 0.12 mm; to −4.64 until −6.2 relative to bregma) using Image J software (ImageJ v1.41, National Institute of Health, Bethesda, MD). Split-cell counting errors were corrected using Abercrombie's formula, where *N* = *n* [*t*/(*t* + *d*)] (*N*: total number of cells; *n*: number of cells counted; *t*: section thickness; and *d*: cell diameter). The total number of cells in the SNc was calculated using Konigsmark's formula, where Nt = Ns (St/Ss) (Nt total number of cells; Ns number of cells counted; St total number of sections through the SNc; Ss number of sections in which cells were counted (Paillé et al. 2007). Image analysis was completed using ImageJ software. The point picker plugin available in ImageJ freeware can be used to mark cells with a colored cross by clicking with the mouse. The number of cells counted is displayed in a text box on completion. For each group of animals, the data were the total number of cells in the section expressed as the percentage of the number of cell bodies measured for the shams (mean ± SD).

Anatomical and behavioral analyses were performed by an experimenter blind to the treatments.

### Locomotor impairment

The rotarod test was performed 2 weeks after surgery. Locomotor impairment was determined on an accelerating rotarod treadmill (TSE systems GmbH, Bad Homburg, Germany). Before testing, the rats were habituated to the instrument at a constant (4 rpm, 5 min) and accelerated speed (40 rpm, 5 min, five times) for three consecutive half days. A testing session was performed on the fourth consecutive day. The rotarod was accelerated progressively from 4 rpm to 40 rpm over 5 min. The time that the rats remained balanced on the device was scored. The rotarod test results for each animal represent the average time (sec) spent on the rod for the last three sequences on the testing session (mean ± SD).

### Statistical analysis

To assess potential differences between groups, a one-way analysis of variance (ANOVA) followed by a Student–Newman–Keuls test were performed. Changes in postoperative measurements over time were analyzed with repeated measurement ANOVA. Paired *t*-tests were used to compare the ipsilateral and contralateral sides. The results are expressed as mean ± SD and differences are considered significant at *P* < 0.05.

## Results

### TH immunohistochemistry

**Figure 1:**
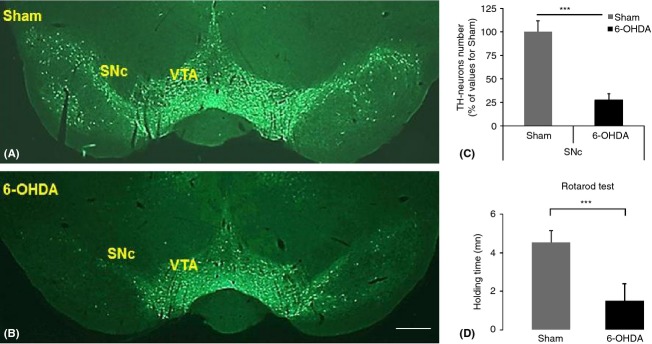
TH immunostaining in sham (A) and 6-OHDA-lesioned animal (B) reveal a drastic decrease in the intensity of staining, mainly in the SNc and less in the VTA. (C) Quantification of TH cells in SNc: the total number of TH-positive neurons was measured in 6-OHDA-lesioned animals (*n* = 6) and shams (*n* = 6) and is expressed as percentage of values obtained in sham rats. Significant differences in the SNc were observed between 6-OHDA-lesioned and sham animals (***P < 0.001). The bar represents 170 *μ*m in A and B. Motor performance (D) was tested by the rotarod at 2 weeks postsurgery and revealed a significant difference in motor disturbance between 6-OHDA-lesioned animals (*n* = 8) in comparison with shams (*n* = 8) (***P < 0.001). mn, minutes.

To analyze the impact of 6-OHDA lesions, TH was explored at the level of SNc (Fig. 1A, B). A considerable decrease in TH staining was observed in the 6-OHDA-lesioned rats (B) compared to the shams (A). This decrease in staining was observed in both the SNc and VTA. The impact of degeneration was measured by counting the TH-positive cells within the SNc, revealing a significant decrease in TH-positive cells in 6-OHDA-lesioned animals (C). Although not counted, a decrease in TH staining within the VTA was also observed and appeared less severe than in the SNc. The TH immunolabeling obtained here is in agreement with previous studies (Paillé et al. 2007; Ouachikh et al. 2013; Zengin-Toktas et al. 2013).

### Locomotor exploration

Locomotor impairment was investigated using the rotarod technique (Fig. 1D) in both lesioned and sham animals. It revealed significant differences in 6-OHDA-lesioned animals (*n* = 8), starting from the first week when compared to the shams (*n* = 8) group (Fig. 1D, week 2: 55 ± 40 sec vs. preoperative stage: 169 ± 25 sec, ***P < 0.001; week 4: 76 ± 36 sec vs. preoperative stage: 169 ± 25 sec, ***P < 0.001; week 6: 95 ± 29 sec vs. preoperative stage: 169 ± 25 sec, ***P < 0.001). Their activity seemed to improve over time but remained different from their preoperative score.

### Dopamine depletion evoked allodynia

**Figure 2:**
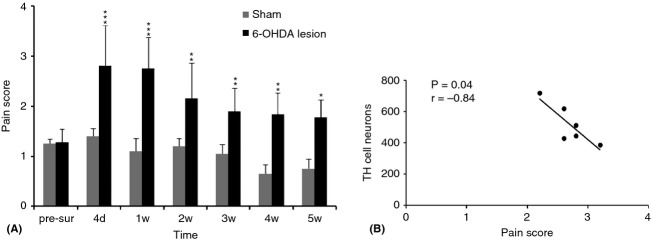
The graph (A) represents the trigeminal pain score as measured by the Vos index in 6-OHDA-lesioned animals (black) and sham (gray). This result was obtained by the application of gentle air puffing on the right side of the infraorbital region. The left side showed the same score for pain. There was a very significant difference between the two groups: 6-OHDA-lesioned and sham groups. This behavioral change is significant up to 5 weeks after surgery. (***P < 0.001, **P < 0.01, *P < 0.05). d, day; w, week. The diagram (B) shows a high inverse correlation (*P* = 0.004) between the total numbers of TH-positive cells in the MDH and the severity of the allodynia. Low TH numbers correspond to high allodynic scores. The slope of the curve is steep (−0.84).

6-OHDA-lesioned rats were assessed for DMA from 4 days to 5 weeks after injury (Fig. 2A). A significant pain score difference was obtained in the 6-OHDA-lesioned animals when compared to the shams. The pain increase was observed in the 6-OHDA-lesioned animals throughout the duration of the experiment. A high inverse correlation (*r* = −0.84; *P* = 0.04) between the allodynic score and the number of TH-positive cells was observed (Fig. 2B). A low TH cell number is correlated with a severe DMA.

### Involvement of PKC*γ*-positive neurons in DMA

**Figure 3:**
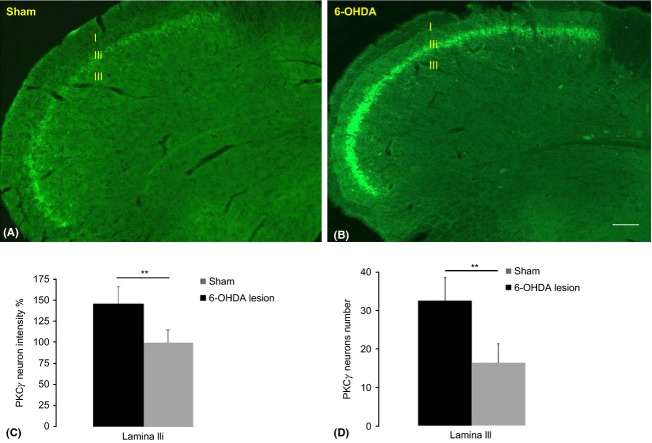
PKC*γ* staining in sham (A) and allodynic MDH (B). Very intense PKC*γ* labeling can be seen in the 6-OHDA-lesioned MDH (B) compared to that of the shams (A). This labeling is observed in the cells of lamina IIi and in scattered cells within lamina III. A very significant difference in PKC*γ* staining can be seen in the MDH lamina IIi of allodynic rats compared to the sham group (C). Also a very significant increase in PKC*γ* cell numbers in lamina III can be observed in the 6-OHDA-lesioned MDH compared to the shams (D). The bar represents 100 *μ*m in A and B, **P < 0.01. I, lamina I.

Next, we examined whether PKC*γ*-positive interneurons are implicated in the DMA in DA depleted rats (Fig. 3). PKC*γ* immunolabeling exhibited a similar pattern of distribution in the sham (Fig. 3A) and 6-OHDA-lesioned (allodynic) rats (Fig. 3B). However, staining intensity had increased bilaterally in the 6-OHDA-lesioned rats in comparison to the shams. Consistent with previous studies (Clifford et al. 1998; Okada-Ogawa et al. 2009), PKC*γ* immunostaining was mainly observed in lamina IIi of the MDH at both the ipsilateral and contralateral sides. Some scattered PKC*γ*-positive cells were also observed within laminae I and outer II (IIo). Careful inspection of PKC*γ* immunolabeling under high magnification confirmed these observations. Interestingly, we noticed a marked increase in both the intensity of staining (Fig. 3B) and the number (Fig. 3C, *P* < 0.001) of PKCγ-positive cells in the MDH lamina III in allodynic animals. The intensity of PKC*γ* staining within lamina IIi was significantly higher in the 6-OHDA-lesioned animal (Fig. 3D) when compared to the shams.

### Mild nonnoxious stimulus induced pERK1/2 expression in MDH laminae

**Figure 4:**
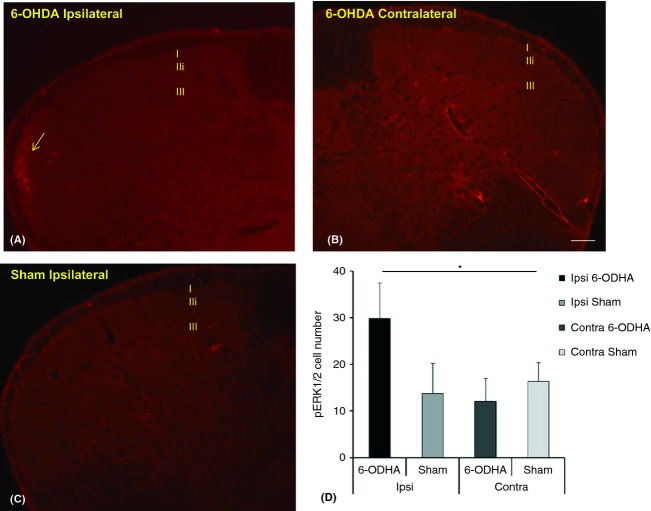
Mild nonnoxious stimulus induced pERK1/2 expression in MDH laminae. In 6-OHDA-lesioned rats (A–B), gentle tactile stimuli induce the expression of pERK1/2 (arrow) in cells located in the ipsilateral superficial laminae I and II. pERK1/2 expression was significantly higher in the 6-OHDA-lesioned MDH when compared to the shams (C). Within 6-OHDA-lesioned MDH, there is a significant increase in the number of pERK1/2-positive cells in the ipsilateral side in response to the stimulus when compared to the contralateral side and sham (D). The bar represents 100 *μ*m, *P < 0.05.

In the 6-OHDA-lesioned rats that received tactile stimuli (Fig. 4), most of the pERK1/2-positive cells were observed in the superficial laminae I and II, in particular in the ipsilateral side receiving the stimulus (Fig. 4A). The superficial lamina II contained the largest number of pERK1/2 immunopositive neurons, which had increased considerably on the ipsilateral side in comparison to the contralateral side (Fig. 4B). Only a very small number of pERK1/2-positive cells were observed in the sham animals (Fig. 4C). Generally, these cells were scattered and located in the whole nucleus and were not restricted to the MDH region that had received a mild nonnoxious stimulation. The number of pERK1/2-positive cells in the ipsilateral superficial lamina of the MDH I of the allodynic rats was significantly higher in comparison to the contralateral or sham rats (Fig. 4D). For laminae III–V, there was no difference between the allodynic rats in comparison to the sham rats regarding the number of pERK1/2-positive cells, ipsilateral or contralateral to the operated IoN. The general pattern of pERK1/2 expression was similar to that obtained previously (Okada-Ogawa et al. 2009).

### PKC*γ* and pERK1/2 are distinct cell subtype

**Figure 5:**
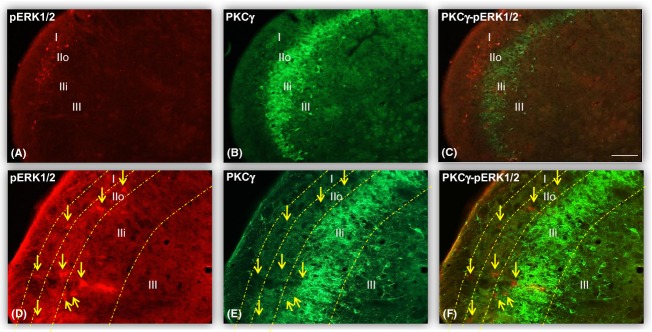
PKC*γ* and pERK1/2 are distinct cell subtypes. Double labeling using pERK1/2 (A and D) and PKC*γ* (B and E) antibodies shows no colocalization (C and F) of either marker within superficial laminae I, II (inner and outer), and lamina III cells. pERK1/2 (arrows) is expressed mostly in superficial laminae I and II (A and D), while a high density of PKC*γ* cells is located in lamina IIi. This result demonstrates distinct pERK1/2 and PKC*γ* cell subtypes. The bar represents 170 *μ*m.

To assess if PKC*γ* and pERK1/2 were present within the same cell, double immunostaining for these markers was performed (Fig. 5A–F). It revealed no colocalization between pERK1/2 (Fig. 5A and D) and PKC*γ* (Fig. 5B and E). These two cell subtypes were distinct (Fig. 5C and F).

### Bromocriptine administration alleviates the DMA

**Figure 6:**
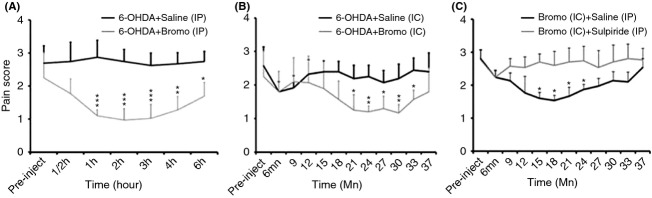
Effects of bromocriptine administration on trigeminal allodynic behaviors in 6-OHDA-lesioned animals. (A) The intraperitoneal administration of 1 mg/kg of bromocriptine induces a significant antinociceptive effect in 6-OHDA-lesioned animals (*n* = 8) when compared to the vehicle-injected group (*n* = 8). This antinociceptive effect lasted up to 4 h postadministration. (B) The intracisternal administration of bromocriptine (1 *μ*g/kg, *n* = 5) significantly decreases the pain score when compared to the vehicle-injected animal (*n* = 5). The effect of bromocriptine was obtained 10 min after its administration and lasted for about 25 mn. (C) Represents the effect of the intracisternal injection of bromocriptine on DMA 90 min after the intraperitoneal administration of sulpiride (D2 receptor antagonist, 50 mg/kg, *n* = 8). Sulpiride administration reverses the effect of bromocriptine on DMA. The bars represent the standard deviation. *P < 0.05; **P < 0.01; ***P < 0.01.

The effect of bromocriptine on pain behavior was investigated in allodynic (6-OHDA-lesioned animals) and compared to allodynic rats injected with saline (Fig. 6). A significant decreased in allodynic behavior was observed after i.p injection of bromocriptine and this effect lasted for 4 h (Fig. 6A).

To demonstrate the local segmental implication of DA in the DMA observed, bromocriptine was administered intracisternally in the 6-OHDA-lesioned animals. Bromocriptine significantly decreased the DMA (Fig. 6B) when compared to animals injected with saline and this effect lasted for 10 min.

#### Sulpiride reversed the analgesic effect of bromocriptine on DMA

Sulpiride, a preferential D2R antagonist, was used to check the specificity of the effect of bromocriptine locally. The intraperitoneal injection of sulpiride (D2R antagonist), 90 min prior to the intracisternal administration of bromocriptine, blocked its effect on DMA (Fig. 6C).

### Bromocriptine administration decreases the expression of PKC*γ* and pERK1/2

**Figure 7:**
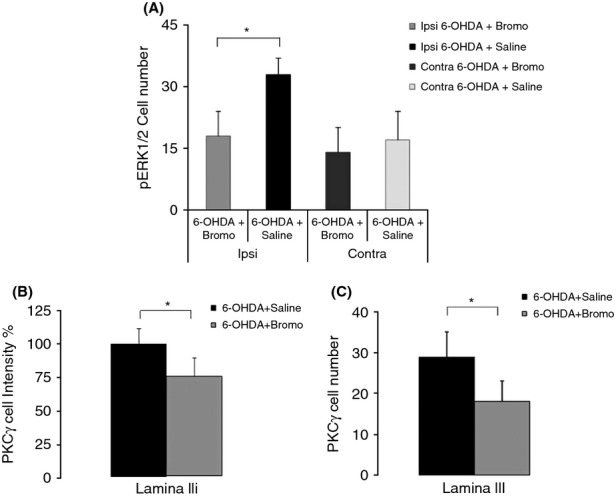
Effects of bromocriptine in pERK1/2 and PKC*γ* expressions. Graph (A) represents pERK1/2 expression after bromocriptine treatment (*n* = 5). It reveals a significant decrease in the number of pERK1/2 cells in the MDH ipsilateral side; however, this decrease is not significant in the contralateral side. PKC*γ* cell expression (*n* = 5) shows a significant decrease in the intensity of staining in lamina IIi (B), and in cell number in lamina III (C). *P < 0.05.

We explored the expression of pERK1/2 and PKC*γ* in the MDH of allodynic and sham animals 3 h after the intraperitoneal injection of bromocriptine (Fig. 7A–C). The rats were anesthetized, stimulated by air puffing, and processed for immunohistochemistry as described in the material and methods section. Bromocriptine administration significantly decreased (*P* < 0.05) the number of pERK1/2 cells within the MDH when compared to the shams (Fig. 7A). However, this decrease was not significant in the contralateral side. Bromocriptine treatment also decreased the intensity of staining in lamina IIi (Fig. 7B) and the number of PKC*γ*-positive cells within lamina III (Fig. 7C).

## Discussion

This study aimed to explore pain as a prevalent nonmotor symptom in PD. To this end, we explored nociception triggered by mild touch stimuli applied to the orofacial region in a rat model of PD induced by 6-OHDA. This is a pioneer study investigating the appearance of trigeminal allodynic behavior in rats with a bilateral dopaminergic lesion of the nigrostriatal pathway. Allodynic behavior was present 4 days after injury and for up to 5 weeks later (experiment duration). The intracisternal and intraperitoneal administration of bromocriptine (D2R-agonist) decreased allodynic behavior in the lesioned animals. Furthermore, the injection of bromocriptine intracisternally, after that of systemic sulpiride, had no effect on the allodynia observed, revealing the clear implication of D2R receptors locally in allodynic behavior. Hence, the antiallodynic effect of bromocriptine was inhibited by D2-type receptor antagonist sulpiride. This result clearly demonstrated the implication of dopamine in trigeminal pain. To support the implication of a segmental mechanism in the neuropathic pain symptom observed in DA depleted animals, the expressions of PKC*γ*, which reveal central sensitization (Malmberg et al. 1997), and those of pERK1/2, which constitute a molecular hallmark of pain sensation (Ji et al. 1999), were explored in the MDH. In this study, we highlight significant upregulation in the expression of both PKC*γ* and pERK1/2 in the MDH of 6-OHDA-lesioned rats when compared to shams. Moreover, the number of pERK1/2-positive cells was significantly higher in the ipsilateral MDH of the stimulated orofacial region when compared to the contralateral nonstimulated region. pERK1/2 was also expressed by cells of lamina I which normally convey nociceptive stimuli. Moreover, these markers were present in distinct cell subtypes. The segmental implication of D2R is also highlighted by the administration of bromocriptine which decreased the number of pERK1/2 cells and the expression of PKC*γ* within the MDH. The present data provided clear evidence of the implication of the dopaminergic nigrostriatal circuitry in the DMA observed in DA-depleted rats and demonstrated for the first time that nonnoxious stimulus can trigger noxious response in rats with a bilateral nigrostriatal pathway lesion. However, we do not exclude the participation of the VTA (mesolimbic and mesocortical pathways; Magnusson and Fisher 2000; Wood 2006; Sogabe et al. 2013) in the DMA as our lesion targeted also this structure.

### Dopamine and nociception

Rats with a bilateral dopaminergic nigrostriatal lesion demonstrated a significant decrease in TH labeling in the VTA, the striatum, and the SNc. Also noted was a significant decrease in the number of TH-positive cells within the SNc, as reported previously (Paillé et al. 2007; Ouachikh et al. 2013; Zengin-Toktas et al. 2013). The bilateral dopamine-depleted animals presented a severe body movement alteration (rotarod) when compared to the controls. These rats were able to move (Paillé et al. 2007; Ouachikh et al. 2013; Zengin-Toktas et al. 2013) and perform all the movements required for the Vos test. Compared to the controls, animals with a dopamine denervated striatum exhibited high sensitivity to nonnoxious stimuli applied to their face. These results are in agreement with a previous study using a unilateral dopamine depletion animal (Chudler and Lu 2008) although the authors reported minor changes in the response to mechanical stimuli. This minor difference between both studies is probably due to the magnitude of the lesion (bilateral vs. unilateral), the nature of the anatomical area lesioned (medial forebrain bundle vs. striatum), and the type of stimuli (static vs. dynamic). This study is also in agreement with previous reports showing that dopamine depletion causes hypersensitivity to mechanical stimulus (Saadé et al. 1997; Takeda et al. 2005). The dopaminergic lesion of SNc enhanced the pain process (decreased threshold and/or latency) in experimental pain tests (Campbell et al. 1988; Morgan and Franklin 1990; Saadé et al. 1997; Altier and Stewart 1999; Takeda et al. 2005; Ansah et al. 2007; Chudler and Lu 2008; Koszewicz et al. 2012). Moreover, pharmacological studies of D2R (agonist/antagonist) in the striatum have reported that it suppresses or enhances the pain process in animal experiments (Magnusson and Fisher 2000; Ansah et al. 2007; Barceló et al. 2010). In addition, systemic use of D2R agonists has proven their antinociceptive action (Michael-Titus et al. 1990; Morgan and Franklin 1990; Clifford et al. 1998). This finding is also supported in this study. These reports clearly demonstrated that D2R has a general antinociceptive effect (Hagelberg et al. 2004). The mechanism by which DA depletion produces neuropathic pain has yet to be determined. To our knowledge, there is no direct projection from SNc to the MDH, therefore we can only explain the nociceptive effect of DA depletion by indirect action on the intermediary descending pain pathway, like that originating from the periaqueductal gray (PAG). The latter constitutes a central structure in the descending pain modulatory pathway (Millan 2002). Previous studies have demonstrated different projections from SNc, SN reticula, VTA, and amygdala to the PAG. One main feature of these projections to the PAG is that they are GABAergic (Rizvi et al. 1991; Cassell et al. 1999; Gauriau and Bernard 2002; Chieng and Christie 2010). DA depletion in these structures may decrease, in one way or another, GABA transmission at the PAG level, hence increasing descending facilitatory pain influences on the MDH. This is supported by the fact that in the 6-OHDA-lesioned animals, Fos expression increased in the PAG after mechanical stimulation or not of the hind paw (Reyes and Mitrofanis 2008). This reflected an increase in neural excitation within the PAG after dopamine depletion. The facilitatory effect of the pain descending pathway is reflected by the increase in PKC*γ* expression within the MDH in this study. PKC*γ* is known to participate in the chronicity of neuropathic pain (Malmberg et al. 1997; Martin et al. 1999; Ohsawa et al. 2001) and PKC*γ* cells are part of a polysynaptic neuronal circuitry which, under disinhibition conditions, induces allodynic behavior (Torsney and MacDermott 2006; Kato et al. 2009; Miraucourt et al. 2009; Takazawa and MacDermott 2010). This polysynaptic circuit contained at least two distincts pERK1/2- and PKC*γ*-expressing cells. The PKC*γ* cells in our condition open the gate, via descending pain facilitation which may induce local disinhibition, thereby allowing tactile nonnoxious stimuli to reach nociceptive processing neurons at the superficial MDH laminae. This is demonstrated here by the expression of pERK1/2, a marker expressed solely by noxious stimuli (Ji et al. 1999). Moreover, pERK1/2 was expressed in this study in the cells of lamina I, which are known to convey nociceptive stimuli. The allodynic behavior observed in dopamine depleted animals is due, at least, to segmental sensitization, as demonstrated by the high increase in PKC*γ* expression and ERK1/2 phosphorylation in the MDH, and its decrease after the i.p. administration of bromocriptine. This sensitization could be the consequence of central dysregulation of the descending inhibitory pain control pathway, hence decreasing its tonic inhibitory action in favor of pain facilitation at the level of the MDH in dopamine depleted rats. This is supported by reports demonstrating that activation of striatal D2R facilitates a descending inhibitory pain pathway (Ansah et al. 2007), and that the activation of striatal NMDA receptors (Pertovaara et al. 2004) provides tonic descending pain facilitatory influences. Indeed, the 6-OHDA depleted rats exhibited an increased concentration of glutamate within the striatum (Meshul et al. 1999; Chassain et al. 2005). Another way in which dopamine depletion may induce MDA is by acting through the prefrontal cortex. The latter constitutes a central structure in the medial pain pathway (Millan 2002). The prefrontal cortex also receives mesocortical dopamine projections which originate from the VTA and a high-frequency stimulation of this structure inhibited nociceptive responses in the rat prefrontal cortex (Sogabe et al. 2013). VTA dopamine depletion could have affected our result by acting on the prefrontal cortex, as the SNc is known not to project to cortical areas.

## Conclusion

This is the first study demonstrating a trigeminal neuropathic process in rats with a bilateral lesion of the nigrostriatal dopaminergic pathway. It also demonstrated the implication of MDH segmental sensitization in the occurrence of DMA. Moreover, the local administration of D2R agonist at the MDH level systemically decreased allodynic behavior. Part of the analgesic effect of bromocriptine occurred at the MDH level and involved D2-type receptors. However, more studies are needed to clarify the neuronal circuitry by which dopamine depletion in the nigrostriatal pathway leads to trigeminal neuropathic pain.
